# Spezifika der Lehre in der Gefäßchirurgie im interdisziplinär-chirurgischen Setting

**DOI:** 10.1007/s00104-022-01636-5

**Published:** 2022-04-11

**Authors:** Udo Barth, Frank Meyer, Zuhir Halloul

**Affiliations:** 1 Klinik für Allgemein-, Viszeral- und Gefäßchirurgie, HELIOS-Klinikum Jerichower Land, Burg, Deutschland; 2 Klinik für Allgemein‑, Viszeral‑, Gefäß- und Transplantationschirurgie, Universitätsklinikum Magdeburg A.ö.R., Leipziger Str. 44, 39120 Magdeburg, Deutschland

**Keywords:** Gefäßchirurgische Lehre, Akademische Lehrstunden, Moderne Lehrmethoden, Exponentieller Wissenszuwachs, Didaktisch kompetente Lehrende, Vascular surgery teaching, Academic teaching hours, Modern teaching methods, Exponential increase of knowledge, Didactically competent teacher

## Abstract

**Ziel:**

Es sollen die komplexen Veränderungen in der gefäßchirurgischen Lehre, Gemeinsamkeiten/Unterschiede zur Allgemeinchirurgie, Spezifika der Lehre/Lehrinhalte sowie deren universitäre Voraussetzungen umrissen werden.

**Methode:**

Kompakte narrative Kurzübersicht.

**Ergebnisse:**

Der gefäßchirurgische Anteil an den chirurgischen Vorlesungen im Universitätsklinikum Magdeburg A.ö.R. umfasst 10 akademische Lehrstunden und beinhaltet die Themen: pAVK, Embolie/Thrombose, Gefäßverletzung, Kompartmentsyndrom, Mesenterialischämie, Aorten‑/Aneurysma- und venöse Chirurgie. Damit liegt die hiesige gefäßchirurgische Lehre deutlich über dem Durchschnitt von 6,1 akademischen Lehrstunden in Deutschland. Die Stärke der (gefäß‑)chirurgischen Ausbildung liegt insbesondere darin, dass der Erkenntnisgewinn am Krankenbett anschließend im Operationssaal insbesondere an visuelle Eindrücke mit simultaner Erklärung gekoppelt werden kann. Eine enge Einbindung der/s Studierenden ins Team ist die hohe Kunst, um die Akzeptanz der (Gefäß‑)Chirurgie als Beruf und Berufung zu fördern. Voraussetzung für eine erfolgreiche Lehre ist ein didaktisch kompetenter Lehrender mit fachlicher Expertise, proaktiver Lehreinstellung, Kenntnissen der speziellen Lernziele, des Curriculums, modernen Lehrmethoden und Bewusstsein für die besondere Vorbildfunktion für Studierende. Die berechtigte und zu stärkende klassische Lehre mittels Vorlesung, Seminar, Praktikum und Lehrbuch wird zunehmend durch Nutzung von internetbasierten Lernplattformen, Bibliotheken und Videoportalen ergänzt.

**Zusammenfassung:**

Die gefäßchirurgische Lehre wird sich in den nächsten Jahren multimodal/-medial mit mehr praxisorientierten Anteilen und intensiver Integration der Studierenden in den Klinikalltag verlagern (müssen).

## Abstract (en)

**Objective:**

The complex changes in vascular surgical teaching and commonalities are to be outlined and discussed, in particular, similarities/differences to general surgery, specifics of teaching/teaching content and its university requirements.

**Method:**

Compact narrative overview

**Results:**

Vascular surgical teaching component at Magdeburg University Hospital comprises 10 academic teaching hours and includes the following topics: PAD, embolism/thrombosis, vascular injury, compartment syndrome, mesenteric ischemia, aortic aneurysm and venous surgery. This puts vascularsurgical teaching here well above the average of 6.1 academic teaching hours in Germany. The strength of (vascular)surgical training lies in the fact that the knowledge gained at the bedside can be linked to visual impressions and simultaneous explanations in the operating theatre. Close integration of the student into the team is the high art of promoting acceptance of surgery as a profession and vocation. The prerequisites for successful teaching are a didactically competent teacher with specialist expertise, a proactive teaching attitude, knowledge of the specific learning objectives, the curriculum, modern teaching methods and awareness of the special role model function for students. Classical teaching by means of lecture, seminar, practical course and textbook is justified and should be strengthened but it is increasingly supplemented by the use of internet-based learning platforms, libraries and video portals.

**Conclusion:**

In the coming years, vascular surgical teaching will (have to) shift to a (rather) multimodal/-media approach with more practice-oriented components and intensive integration of students into everyday clinical practice.

## Hintergrund

Im Zuge

- der zunehmenden Bürokratie in der täglichen Stationsroutine,
- des auch in den Universitätskliniken merkbaren Nachwuchsmangels,
- der zunehmenden Komplexität der Erkrankungen als Folge des demographischen Wandels und
- der deutlichen Zunahme alter und multimorbider Patienten sowie nicht zuletzt
- des wachsenden Fortschritts des medizinischen Wissens und dessen immer schneller werdender Verbreitung durch neue Medien

ist es ungleich schwerer geworden, eine den aktuellen Bedürfnissen der Studierenden angepasste Lehre und Ausbildung zu gewährleisten. Auch in der schulischen Ausbildung wird durch die Lehrerschaft eine Veränderung der Wissensaneignung in den neuen Generationen bemerkt. So lässt sich heutzutage die Studiererendenschaft nicht mehr mit althergebrachten Lehransätzen zufriedenstellen. Um weiter eine gute Ausbildung zu gewährleisten und einen niveauvollen Nachwuchs zu generieren, sollte die studentische Lehre immer wieder den Bedürfnissen und Erfordernissen der aktuellen Zeit angepasst und modernisiert werden. Dies betrifft gerade die chirurgischen Fächer, da die Lehrenden zum großen Teil der Arbeitszeit fest im Operationssaal gebunden sind und im Zeitablauf wenig flexibel sind. Doch liegen auch hier besondere Chancen, da gerade in der Chirurgie die Möglichkeit besteht, das erlernte Wissen unmittelbar in ein visuelles Erleben, begleitet von zeitnaher Erklärung, umzuwandeln und den Studenten mit in das Erfolgserlebnis einer Operation einzubeziehen.

In dieser Übersicht sollen die komplexen Veränderungen, Möglichkeiten und Perspektiven der Lehre im Fachgebiet Gefäßchirurgie anhand von Gemeinsamkeiten und Unterschieden zur Allgemeinchirurgie, Spezifika der Lehre und Lehrinhalte sowie deren universitäre Voraussetzungen umrissen werden.

## Material und Methoden

Kompakte narrative Kurzübersicht, basierend auf selektiven Referenzen der aktuellen medizinisch-wissenschaftlichen Literatur und eigenen klinisch-didaktischen Lehrerfahrungen aus der täglichen Praxis des Fachgebiets Gefäßchirurgie.

## Ergebnisse (Eckpunkte)

### Geschichte der Lehre in der Gefäßchirurgie

Seit dem Barockzeitalter entwickelte sich langsam die Lehre für die Chirurgie an den Universitäten. Die Chirurgen galten aber weiterhin nur als Heilkundige zweiter Klasse. Davor fand die Ausbildung zum Chirurgen an den Hochschulen keine Berücksichtigung, da die mehr praktische Ausbildung des Chirurgen eine hauptsächlich handwerkliche Orientierung habe und wissenschaftlicher Ballast nicht erforderlich sei. Erst die Einrichtung chirurgischer Lehranstalten führte zu einer Verbesserung der Qualität und Quantität. Die wachsende Spezialisierung erforderte die Wiedereingliederung der Chirurgie in die Medizin, sodass sich die Chirurgie um die Mitte des 19. Jahrhunderts einen Platz im Universitätsunterricht eroberte [1]. Erst nach der Gründung des Deutschen Reiches 1871 war die Ausübung des Chirurgenberufes an ein Medizinstudium geknüpft [2]. Zudem wurde der klinische Unterricht kontinuierlich ausgeweitet und die erste reichseinheitliche Prüfungsordnung mit der Bekanntmachung vom 25.09.1869 „betreffend die Prüfung der Ärzte, Zahnärzte, Thierärzte und Apotheker“ festgelegt. Voraussetzung für die Zulassung zur Staatsprüfung war ein 4‑jähriges Medizinstudium mit Abgangszeugnis der Universität und Ablegen der Vorprüfung im 5. oder 6. Studiensemester [3]. Die erste Facharztordnung in Deutschland wurde 1924 auf dem Deutschen Ärztetag in Bremen beschlossen [4]. Die Entwicklung der rekonstruktiven Gefäßchirurgie beginnt Ende des 19. und Anfang des 20. Jahrhunderts. In den 70er-Jahren des 20. Jahrhunderts löste sich die Gefäßchirurgie als eigenständiges Fachgebiet aus der Chirurgie und trug zunehmend Verantwortung für die Lehre gefäßchirurgischer Erkrankungen und deren Behandlung.

Mit der drastischen Erhöhung der Anforderungen an Ärztinnen und Ärzte im letzten Drittel des 20. Jahrhunderts durch exponentielles Wachstum des medizinischen Spezialwissens, des technologischen Fortschritts, veränderte Rahmenbedingungen der Gesundheits- und Sozialpolitik sowie den demographischen Wandel wurde es notwendig, die ärztliche Aus- und Weiterbildung zu überarbeiten und ein Absolventenprofil zu definieren [2]. So ist es zum Ziel geworden, die reine Faktenvermittlung zur kompetenzbasierten Ausbildung umzuwandeln. So kam es zur Einführung des „Nationalen kompetenzbasierten Lernzielkatalogs Medizin“ (NKLM) im Jahr 2015. Hier wurden kompetenzbasierte Lernziele für die Gefäßchirurgie im Studium definiert, u. a. die selbständig symptombezogene Anamnese und systematische klinische Untersuchung des Gefäßstatus, die Planung der weiterführenden Diagnostik und Therapie, basierend auf dem Untersuchungsbefund und selbständige sonographische Darstellung der Aorta und des „Venensterns“ sowie der Benennung der sonographischen Kriterien für ein Aortenaneurysma und der tiefen Venenthrombose [5].

### Gemeinsamkeiten zwischen chirurgischen Fächern

Die chirurgischen Vorlesungen in der universitären Ausbildung werden in der Regel im Verbund abgehalten, wobei die gefäßchirurgischen Themen mit einem Anteil von 10 akademischen Lehrstunden durch Dozenten der Gefäßchirurgie referiert werden. Die Lehrstunden beinhalten folgende Themen:

- pAVK (periphere arterielle Verschlusskrankheit),
- Aortenchirurgie,
- Aneurysmachirurgie,
- Embolien,
- Mesenterialischämie,
- venöse Chirurgie,
- Gefäßverletzungen,
- Kompartmentsyndrom,
- chronische Wunden.

Damit liegt die hiesige gefäßchirurgische Lehre deutlich über dem Durchschnitt von 6,1 akademischen Lehrstunden in Deutschland [6]. Seminare und Blockpraktika werden einheitlich organisiert und sind im Wesentlichen von der Lehre am Krankenbett und der unmittelbaren Assistenz im Operationssaal geprägt. Hier liegt gerade die Stärke der chirurgischen Ausbildung, dass der Erkenntnisgewinn am Krankenbett anschließend im Operationssaal an visuelle Eindrücke inklusive simultaner oder zumindest zeitnaher Erklärung gekoppelt werden kann, was in konservativ agierenden Fächern selten der Fall ist. Dies setzt sich im Besonderen im Praktischen Jahr (PJ) fort, wo dieser Effekt kontinuierlich über das Tertial erreicht werden sollte. Hier ist gerade eine enge Einbindung der „PJlerinnen“ und „PJler“ in das chirurgische Team die hohe Kunst, um die Akzeptanz der Chirurgie als Beruf und Berufung zu fördern. Das perioperative Management mit Indikationsstellung in der Sprechstunde, Operationsaufklärung, Operationsvorbereitung, stationärer Betreuung (perioperatives Management) und Entlassungsmanagement ist im wesentlichen Ablauf einheitlich und kann von den Studierenden schnell erlernt und mitbetreut werden. Der Erwerb spezifisch chirurgisch-motorischer Fähigkeiten durch Hand-Auge-Koordination, Wiederholung und Training kann in extrakurrikularen Aktivitäten gefördert werden [7].

Hier haben in den letzten Jahren verschiedene Möglichkeiten der extrakurrikularen Aktivitäten Eingang in den Lehralltag gefunden. Zu nennen sind hier das Mentorenprogramm, in dem sich der Mentor der Gefäßchirurgie für junge Studenten einsetzt, sie über die Studienjahre betreut und berät sowie Unterstützung bei Famulaturen, Auslandsaufenthalten, Promotionsarbeiten und Fachweiterbildungen anbietet. Des Weiteren bietet die „Deutsche Gesellschaft für Gefäßchirurgie und Gefäßmedizin (DGG) – Gesellschaft für operative, endovaskuläre und präventive Gefäßmedizin e. V.“ ein Programm zur Förderung des gefäßchirurgischen Nachwuchses (MAGiC) an. Hierfür wird auf den Jahrestagungen der DGG ein eigens entwickeltes Kongressprogramm für Stipendiaten angeboten, das den MAGiC-Teilnehmenden einen tieferen Einblick in das Fach geben soll. Auch zu erwähnen ist die Etablierung von Trainings- und Übungslaboren, sog. „Skills Labs“ an den Universitäten [7]. Hier können in gefäßchirurgischen Modulen, die teils auch schon wegen erkannter und zur festen Etablierung eingestufter Relevanz kurrikular eingeführt worden sind, z. B. die grundlegenden Fertigkeiten der Gefäßbeurteilung mittels manueller und dopplersonographischer Techniken erlernt und trainiert werden.

### Unterschiede zwischen den chirurgischen Fächern

Die Gefäßchirurgie unterscheidet sich von der Allgemein- und Viszeralchirurgie zunächst einmal in der Patientenklientel. Gefäßchirurgische Patienten leiden an einer Systemerkrankung, die über eine einseitige gefäßchirurgische Betrachtungsweise weit hinausgeht und die sich vielfältig in verschiedenen Organsystemen widerspiegelt und nicht selten multimorbide Patienten hervorruft. Hier ist ein breites Wissen auch in den Fachdisziplinen Angiologie, Kardiologie, Nephrologie, Neurologie, Gastroenterologie, Diabetologie und Endokrinologie gefragt. Um die fachübergreifende Betrachtungsweise zu erlernen, bedarf es neuer Wege in der Ausbildung der Studierenden. Was der Pädagoge als „vernetzten Unterricht“ kennt, ist in der praktischen studentischen medizinischen Lehre noch relativ unterentwickelt. Während die Intensivstationen häufig interdisziplinär geführt werden, sind begleitende interdisziplinäre Visiten auf den Normalstationen eine Seltenheit. Etabliert dagegen haben sich bereits interdisziplinäre Fallkonferenzen unter Beteiligung der internistischen Teilgebiete und Radiologie unter der Führung der Gefäßchirurgie. Mit zunehmender Verschmelzung der Angiologie und Gefäßchirurgie zur Gefäßmedizin ist in den nächsten Jahren eine zunehmende Vernetzung der Gefäßchirurgie mit den Teildisziplinen der Inneren Medizin auch in der Lehre zu erwarten. Dies bietet zudem auch die Gelegenheit, die Facharztweiterbildung durch interdisziplinäre Kompetenzmodule zu erweitern, da das Problem der Multimorbidität hier bisher zu wenig Berücksichtigung findet.

Die zunehmende Verwendung endovaskulärer Verfahren als „First-line“-Therapie bietet dem Lernenden die Möglichkeit, die Gefäßchirurgie auch visuell besser zu verstehen. So sollten gleichrangig zu den Nahtkursen auch Trainingsmodule angeboten werden, in denen grundlegende Techniken und Basiswissen über endovaskuläre Materialien gelehrt und manuelle Fähigkeiten, wie z. B. sonographiegestützte Punktion, Drahtführung, Schleusen- und Katheterhandhabung sowie die Benutzung eines Manometers (und ggf. auch initial der simplen Prothesenfreisetzung – zumindest im Demonstrationsmodus) erlernt werden.

Die Durchführung einer gefäßchirurgischen Operation erfordert zudem eine andere Sichtweise auf den Operationsverlauf, d. h. dass die vaskuläre Rekonstruktion eine Diversität von Rekonstruktionsverfahren bietet, die häufig durch deren nacheinander geschaltete Anwendung bis zum gewünschten Endergebnis charakterisiert ist. Zudem ist die Kombination der offenen und endovaskulären Therapieverfahren als Hybrideingriff eine gute Möglichkeit, die Therapie auf den einzelnen Patienten abzustimmen. Dies erfordert ein breites Wissen und Können des Operateurs in beiden Sparten, sodass der Wissensgewinn des Lernenden bei gefäßchirurgischen Operationen insgesamt sehr hoch ist. So ist der Einsatz von Studierenden als zweitem oder drittem Assistent zur praktischen Wissensvermittlung sehr wertvoll und aussichtsreich.

### Voraussetzungen/Anforderungen für Lehre

Das Hochschulgesetz des Landes Sachsen-Anhalt (HSG LSA) in der Fassung der Bekanntmachung vom 14.12.2010 regelt in Abschnitt 2 das Studium und die Lehre. Als Ziele werden formuliert:

„Lehre und Studium sollen die Studierenden auf berufliche Tätigkeiten vorbereiten und ihnen die erforderlichen fachlichen Kenntnisse, Fähigkeiten und Methoden für den jeweiligen Studiengang so vermitteln, dass sie zu wissenschaftlicher oder künstlerischer Arbeit, zu selbstständigem Denken und verantwortlichem Handeln in einem freiheitlichen, demokratischen und sozialen Rechtsstaat befähigt werden. Lehre und Studium sollen die Grundlage für berufliche Entwicklungsmöglichkeiten und für die Fähigkeit zur eigenverantwortlichen Weiterbildung schaffen. Die Hochschulen gewährleisten, dass die Studierenden dieses Ziel im Rahmen der jeweils geltenden Regelstudienzeit erreichen können.“

Zur Qualität der Lehre wird lediglich in § 7 erläutert:

„Die Hochschulen ergreifen die notwendigen Maßnahmen zur Qualitätssicherung in der Lehre. Die Qualität der Studienangebote sichern die Hochschulleitungen und die Dekaninnen und Dekane im Rahmen ihrer Zuständigkeit insbesondere durch Lehrevaluationen gemäß Absatz 2 und durch Verfahren zur Sicherung und Entwicklung der Qualität in Studium und Lehre nach § 7a.“

Auch der § 8 zur Studienreform formuliert nur grob, dass

„im Zusammenwirken mit dem Ministerium Inhalt und Form des Studiums im Hinblick auf die Entwicklung in Wissenschaft und Kunst, die Bedürfnisse der beruflichen Praxis und die notwendigen Veränderungen in der Berufswelt zu überprüfen und weiterzuentwickeln sind.“

So sollen die Studieninhalte den Studierenden im Hinblick auf Veränderungen in der Berufswelt breite berufliche Entwicklungsmöglichkeiten eröffnen, die Formen der Lehre und des Studiums den jeweils fortgeschrittenen methodischen und didaktischen Erkenntnissen entsprechen und die Studierenden befähigt werden, wissenschaftliche oder künstlerische Inhalte sowohl selbständig als auch im Zusammenwirken mit anderen zu erarbeiten und deren Bedeutung für die Gesellschaft und die berufliche Praxis zu erkennen [8]. Vertiefend regelt dann die Approbationsordnung für Ärzte in § 2 die Unterrichtsveranstaltungen, deren Ziele und Durchführung. Hier ist besonders hervorzuheben, dass die Vermittlung des theoretischen und klinischen Wissens während der gesamten Ausbildung so weitgehend wie möglich miteinander verknüpft wird. Im Weiteren regelt hier die Approbationsordnung z. B. auch die Stundenzahl der Unterrichtseinheiten, die Höchstzahl der Studierendengruppen und die Unterrichtsstunden der Blockpraktika [9]. Im speziellen gefäßchirurgischen Fall wurde an Universitäten mit Ordinariat oder eigenständiger gefäßchirurgischer Klinik mit 7,0 bzw. 7,2 h im Semester im Durchschnitt 2 h mehr gelehrt als an Universitäten, in denen die Gefäßchirurgie als Sektion oder sonstige Organisationseinheit vertreten ist (5,4/5 h). Dieser Unterschied war statistisch allerdings nicht signifikant [6]. Das heißt aber, dass nur eine Aufwertung der Gefäßchirurgie durch strukturelle Veränderungen zu einer Ausweitung der gefäßchirurgischen Lehre an den Universitätskliniken führt [6].

Voraussetzungen für eine erfolgreiche Lehre ist

- ein didaktisch kompetenter Lehrender mit
  - fachlicher Expertise,
  - proaktiver Einstellung zur Lehre,
  - Verständnis der grundlegenden didaktischen Grundprinzipien,
  - Kenntnissen
    der speziellen Lernziele,
    des Kurrikulums,
    über Lehrmethoden (und)
  - sukzessiven Lehrerfahrungen (sowie)
  - Bewusstsein für die besondere Vorbildfunktion für Studierende [5].

Der Nachweis einer Didaktikschulung sowie über die Durchführung von Lehrveranstaltungen ist in der Habilitationsordnung gefordert. Ein weiteres Instrument der didaktischen Professionalisierung stellt das dreistufige „Train-the-Trainer“-Konzept mit Basiskurs, Aufbaukurs und Masterclass dar. Ziel ist die Vermittlung moderner Lehrmethoden und Strategien, die dem klinisch Lehrenden ermöglichen soll, eine effektive Lehre trotz hoher Arbeitsbelastung im Alltag zu gestalten [10]. Vor dem Hintergrund der Veränderungen durch die neue Ärztliche Approbationsordnung 2003 wurde 2004 ein postgradueller und interfakultärer „Master-of-Medical-Education“-Studiengang unter Schirmherrschaft des „Medizinischen Fakultätentages“ geschaffen, der Multiplikatoren und Führungspersonen in der medizinischen Ausbildung qualifiziert, ein deutschsprachiges Netzwerk aufbaut und die Lern- und Ausbildungsforschung stärken soll [11]. Dieser Studiengang wird von der Medizinischen Fakultät Heidelberg zusammen mit sechs weiteren deutschen medizinischen Fakultäten angeboten und gibt einen umfangreichen Einblick von der Kurrikulumsentwicklung bis zur Evaluation einer medizinischen Ausbildungsstätte.

Die Lehre im Rahmen der klinischen Routine stellt eine besondere Herausforderung dar. Hier wird die Lehre auch an die Vertreter des Lehrstuhlinhabers weitergegeben, denn die Lehre ist integraler Bestandteil der Beschäftigung an einer Universitätsklinik. Jedoch muss auch die Klinikleitung den lehrenden Vertretern entsprechende Freiräume schaffen, da meist die klinische Versorgung im Vordergrund steht [6] und durch Dienste, Dienstabgeltung und Personalmangel die zu Verfügung stehenden Ressourcen meist knapp bemessen sind.

Ein Weg aus diesem Problem könnten neue Qualifizierungswege in der Universitätsmedizin sein, die wissenschaftliche und klinische Laufbahn trennen. So liegt bei der wissenschaftlichen Laufbahn der Schwerpunkt auf Forschung und Lehre, während die Vertreter der klinischen Laufbahn den Schwerpunkt auf der Krankenversorgung haben [12], was zu einer weiteren Professionalisierung in beiden Bereichen führen könnte. Dies erfordert jedoch eine institutionell enge Kooperation und Organisation, um Dissoziierungstendenzen zu überbrücken [12].

Effektive Lehre im klinischen Umfeld wird häufig durch mangelnde Planung, unzureichende Anleitung und Supervision sowie fehlendes oder falsch durchgeführtes Feedback für die Lernenden behindert [3]. Die Herausforderung wird zudem in der Lehre der praktischen Fertigkeiten verstärkt. Hier können extrakurrikulare Aktivitäten die Lehre praktischer Fähigkeiten verbessern, was jedoch eine hohe Motivation der Lehrenden und Lernenden voraussetzt [7]. Die klassische Lehre mittels Vorlesung und Lehrbuch wird zunehmend durch Nutzung von internetbasierten Lernplattformen, Bibliotheken und Videoportalen ergänzt, was einerseits die Anpassung des Lernens an die individuellen Bedürfnisse erlaubt, andererseits die Akzeptanz der klassischen Lehre verringert. Zudem ist der Inhalt der Internetquellen in der Regel nicht überprüft, da keine oder nur selten eine Qualitätskontrolle durch ein Fachgremium existiert.

### Lehrinhalte

**Figure 1:**
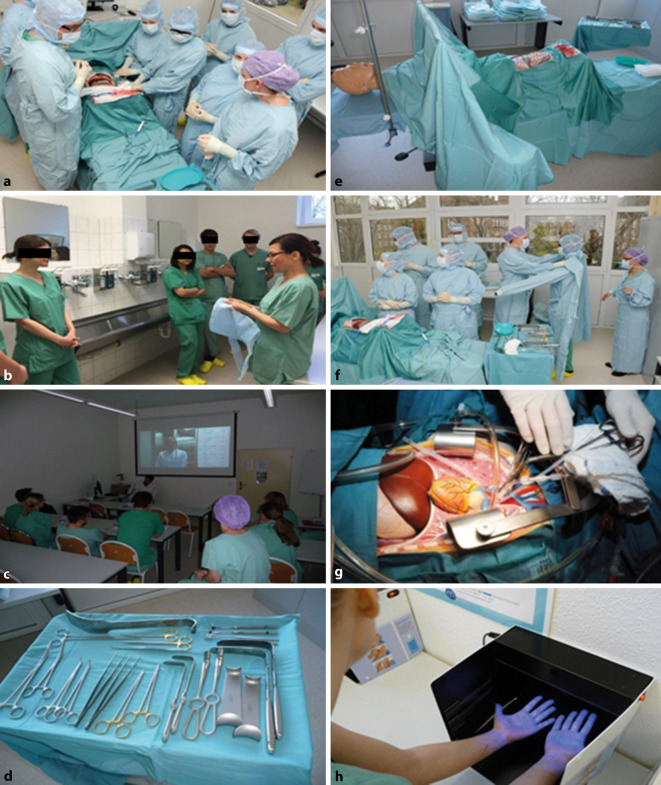

Diese werden – ausgehend vom NKLM und der „Ärztlichen Approbationsordnung“ (s. oben) – durch folgende Eckpunkte bestimmt: pAVK, Aortenchirurgie, Aneurysmachirurgie, Embolien, Mesenterialischämie, venöse Chirurgie, Gefäßverletzungen und das Kompartmentsyndrom sowie die chronische Wunde. Die Wissensvermittlung erfolgt mittels kurrikularer Veranstaltungen wie Vorlesungen, Seminare und Blockpraktika (Abb. 1 und 2) und das „problemorientierte Lernen“ (POL) sowie fakultativer, wie beispielsweise individueller „Bed-side-teaching“-Termine/-Veranstaltungen oder (prüfungsvorbereitender) Repetitorien, aber auch durch das unersetzliche Selbststudium (Tab. 1**)**. Das problemorientierte Lernen (POL) ist eine didaktische Methode zur gezielten Erarbeitung von Lerninhalten in kleinen Gruppen, bestehend aus einer Kombination von Gruppendiskussionen und Selbststudium. Ein(e) DozentIn fungiert hier als ModeratorIn, der die Kleingruppenarbeit in zunehmender Eigenverantwortung der Lernenden anleitet. Dadurch erhält der Lernende die Möglichkeit, die Lernziele aktiv mitzugestalten, wodurch die Motivation erwachsener Lernender deutlich zunimmt. Insbesondere im chirurgischen Praktikum bestehen dadurch eine höhere Lernmotivation, aktivere Mitarbeit und bessere Gesamtbeurteilung der Lehrveranstaltung [13].

**Figure 2:**
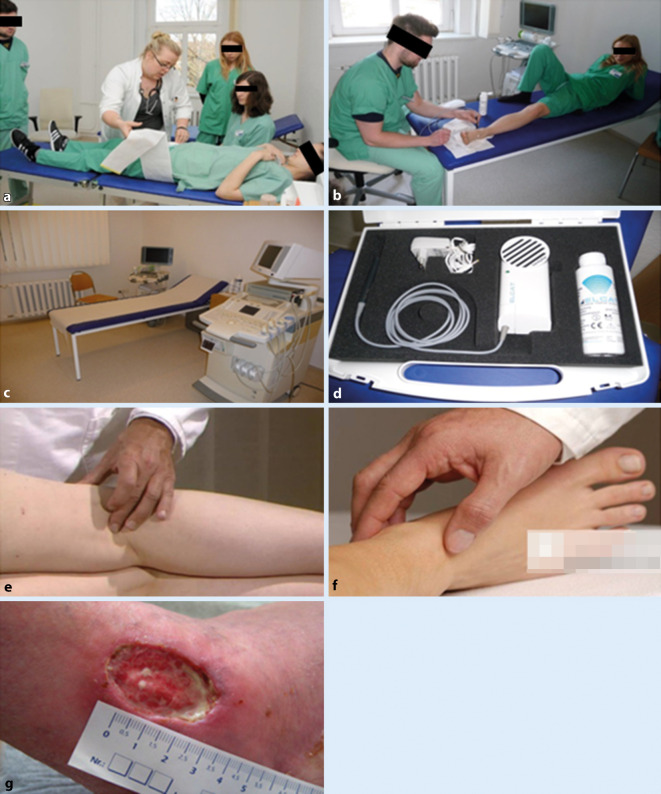

**Table 1:** 

Lehrbestandteil	Spezifika in der Gefäßchirurgie
Vorlesung	pAVK
	Aneurysmachirurgie
	Aortenchirurgie
	Embolien
	Venöse Chirurgie
	Kompartmentsyndrom
	Mesenterialischämie
	Gefäßverletzung
	Chronische Wunde
Seminare/TPF	*Klassifikation der pAVK*
	*Erwerb Kenntnisse Gefäßdiagnostik* Pulsstatus Grundkenntnisse der Doppler- und Duplexsonographie Erhebung Knöchel-Arm-Index
	*Beurteilung und Behandlung chronischer Wunden* Wundphasen Stadiengerechte Wundbehandlung Verbands- und Wundmaterialien
	*Anlage von Druck- und Kompressionsverbänden*
Famulatur	Gefäßmedizinisch spezifische Anamneseerhebung und Untersuchung
	Erste Erfahrungen der Doppler- und Duplexsonographie
	Visualisierung der Revaskularisationsbemühungen und -ergebnisse im Operationssaal mithilfe der DSA
PJ	*Neben den allgemeinen PJ-Ausbildungsinhalten und -zielen (nach Vorgabe des einrichtungsspezifischen PJ-BASIS-Logbuchs des Medizinischen Fakultätentages) sollte die PJlerin/der PJler* Einen Wundbehandlungsplan für akute und chronische Wunden erstellen Einen vollständigen und korrekten peripheren Pulsstatus erheben und dokumentieren Die orientierende Untersuchung hinsichtlich der Thrombose, der venösen Insuffizienz, der Polyneuropathie sowie der chronischen und akuten Ischämie (inkl. orientierender Klassifikationen) anwenden
	*Folgende gefäßchirurgische Krankheitsbilder sollten z.* *B. erlebt und dokumentiert werden* pAVK Stadium II–IV Diabetischer Fuß Arterielle Thrombembolie Aortenaneurysma Kompartmentsyndrom

Die o. a. inhaltlichen Eckpunkte der „speziellen Chirurgie“ erlauben ideal den Bezug zu Basisthemen der „allgemeinen Chirurgie“ wie prinzipielle Operationstechniken, Blutungen, Embolien/Thrombosen, Wunden etc., die daher nicht selten gerade von gefäßchirurgisch aktiven Operateuren und Lehrkräften mit besetzt sind und in der Lehre meist aufgrund der verinnerlichten und gelebten und damit auch kompetent vermittelten Praxisrelevanz durchweg sehr erfolgreich abgehalten werden.

### Vorlesung

An vorderster Stelle der Wissensvermittlung steht auch heute noch die Frontalvorlesung, die in der direkten Weitergabe des Wissens an die Studierenden ihre Bedeutung nicht verlor – im Gegenteil. Im Zuge der SARS-CoViD-19-assoziierten Konsequenzen mit zeitweise dominierenden ausschließlichen onlinebasierten Lehrveranstaltungen erlebte die Frontalvorlesung geradezu eine Renaissance.

*Vorteil* der Vorlesung ist die Möglichkeit der Verdichtung des Wissens auf die notwendigen Kernpunkte zur weiteren Studienanleitung und Vertiefung in die Materie im Selbststudium in einer einzigartig effektiven Lehrform (1 DozentIn: ≥ 500 Studierende; gerade bedeutsam für die gegenwärtigen Personalverhältnisse) mit dem notwendigermaßen – aufgrund der (heraus-)fordernden Lehrform – ehrlichen Bekenntnis des Lehrenden hinsichtlich seiner wirklichen Lehrhaltung und zwangsläufig unverkennbaren Persönlichkeit. Ein weiterer unstrittiger Vorteil ist die fast unendliche Option der Studierenden, sich im Hören und Erfassen aufgrund der Verdichtung prägnanter Inhalte mit wissenschaftlichem Hintergrund zu üben (das den wenigsten „in die Wiege gelegt ist“). Aufgrund der fast täglichen Erweiterung des medizinischen Fachwissens und stetigen Aktualisierung der Evidenz in kürzerer Zeit kann es dem ungeübten Lernenden allerdings schwerfallen, hier die entsprechenden Schlüsse zu ziehen und sich nicht in der Vielfalt der Publikationen und medialen Angebote zu verlieren. Auch ist es hier möglich, das Wissen anhand klinischer Beispiele visuell und auditiv in direktem Kontext zum Thema zu untermauern.

Der *Nachteil* einer Vorlesung liegt zunächst in der Abhängigkeit der Qualität der Wissensvermittlung von den didaktischen Fähigkeiten des präsentierenden Dozenten, weiter in dem Lehr- und Präsentationstempo, welches die individuellen Bedürfnisse der Studierenden ignorieren könnte, da hier unterschiedliche Lerntempi und Lernstrategien vorhanden sind [14]. Daher sollte sich die chirurgische Vorlesung an die multimedialen und vernetzenden Möglichkeiten anpassen und erneuern, z. B. durch vorlesungsbegleitende Webseiten und Lernplattformen, um der abnehmenden Zahl der Vorlesungsbesucher entgegenzuwirken [14].

### Seminare/TPF

Die klassischen Seminare sind im Wesentlichen den Blockpraktika und der kurrikularen Lehre im „Skills Lab“ gewichen. Der von den Studierenden häufig bemängelte unzureichende Erwerb praktischer Fähigkeiten [15] und der mangelnde Patientenkontakt, um das gelernte Wissen anzuwenden, machte die Konzentration der praktischen Studierendenseminare in diesen Formen notwendig. In Vorbereitung auf das Blockpraktikum dient das einheitliche „Training praktischer Fertigkeiten“ (TPF), wo chirurgische Basisfertigkeiten vermittelt werden, sodass die Studierenden ohne Druck praktische Fertigkeiten erlernen können, um sie dann in der Praxis mit mehr Sicherheit und Kompetenz anwenden zu können [16].

Das TPF in der Gefäßchirurgie umfasst den Erwerb von Kenntnissen in der Gefäßdiagnostik. Dabei werden die manuellen Fähigkeiten in der Erhebung des Pulsstatus trainiert sowie die Grundkenntnisse in der Doppler- und Duplexsonographie vermittelt. Praktische Übungen zum Umgang mit dem „Hand-Doppler“ und Basiseinstellungen am Ultraschallgerät (Abb. 2) geben erste Einblicke in die tägliche Routine des Gefäßchirurgen (in beabsichtigter Brücke zum interdisziplinären Partner, zur Angiologie). Wichtig ist hierbei die Erhebung des Knöchel-Arm-Index („ankle-brachial index“, ABI) und dessen Beurteilung zur Bewertung der pAVK, die Beurteilung der dopplersonographischen Flusskurven, die einen Hinweis auf die intravasalen Flussverhältnisse geben. Die Rekapitulation der arteriellen und venösen Gefäßversorgung des Körpers festigt zudem das Wissen um die häufigsten Erkrankungspunkte im Gefäßsystem. Ein weiterer Punkt in der Ausbildung der Studierenden ist die Beurteilung und Behandlung chronischer Wunden. Die Beurteilung der Wundphasen ist die Voraussetzung für eine stadiengerechte Wundbehandlung, sodass bei der Vielfalt der modernen Verbands- und Wundmaterialien die chronische Wunde effektiv und kostengünstig behandelt werden kann. Das Anlegen von Druck- und Kompressionsverbänden kann ebenfalls mit wenig Aufwand mit den Studierenden geübt werden. Im anschließenden Blockpraktikum können das erworbene Wissen und die erworbenen Fähigkeiten in der gefäßchirurgischen Stationsroutine direkt umgesetzt werden. Hier gilt es, das erworbene Wissen zum Teil auch anzuwenden. Daher ist auch im Blockpraktikum die Aufnahmeuntersuchung ein probates Mittel, um die Kenntnisse der Basisgefäßdiagnostik und der Wundbeurteilung anzuwenden. Selbstverständlich ist hier die Überprüfung der Befunde und Dokumentation durch den Stationsarzt zu fordern. Wünschenswert wäre es, wenn der Student den Patienten am darauffolgenden Tag auch mit in den Operationssaal begleitet, um die direkten Konsequenzen seiner Untersuchungen zu begreifen. Die im Skills Lab geübten Verfahren der Hautnaht sollten zudem unter Anleitung gefestigt werden. Der Abschluss des Abschnitts von TPF und Blockpraktikum bildet eine schriftliche Klausur, die einerseits das erworbene Wissen des Studenten abfragt, andererseits die Vermittlungsfähigkeit der Lehrenden nüchtern prüft und objektiv erfasst.

Die Erfahrungen der Studenten im Skills Lab mit dem Magdeburger Modell werteten Piatek et al. im Rahmen einer Kursevaluation des chirurgischen Naht- und Knüpfkurses im Wintersemester 2012/2013 und Sommersemester 2013 aus. Bei einer Übungszeit von 90 min sowie Naht- und Knüpfübungen an Silikonnahtkissen und Knüpfbrettern stimmten nur 37 % der Studenten zu, dass die Übungszeit ausreiche. Zudem wurde der Wunsch geäußert, an Schweinefüßen die Nahttechniken zu üben. Nach einer räumlichen und technischen Umstrukturierung konnte die praxisnahe Übungsvariante mit Schweinefüßen umgesetzt und die Kurszeit verdoppelt werden. Im Wintersemester 2013/2014 zeigte sich dann, dass die Lerninhalte für 100 % der Studierenden klar formuliert und die Darstellung der Lerninhalte für 97,2 % verständlich war. Für die Mehrheit war nun die Kurszeit ausreichend. Vor dem Kursbeginn gab keiner der Studenten an, sich „völlig sicher“ und „sicher“ zu fühlen, was nach dem Kurs zu 62,2 % jeweils der Fall war [15].

### Famulatur

Die Famulatur ist der erste längerfristige und kontinuierliche Kontakt der Studierenden mit dem chirurgischen Klinik‑, Stations- und Arbeitsalltag. Hier entstehen wahrscheinlich die ersten Prägungen für die spätere Wahl der Fachrichtung der Studierenden und diese sollten daher genutzt werden, das Interesse an dem Fachgebiet zu wecken. Es ist natürlich schwierig, das Fachgebiet nur von seiner „Schokoladenseite“ aus zu zeigen, jedoch sind erfahrungsgemäß die besten Erfolge durch Integration der Studierenden in Diagnostik und Therapie (eben in die Operation) mit kontinuierlicher Erläuterung von Hintergrundwissen zu erzielen. In der Gefäßchirurgie eignen sich besonders Instruktionen während der Duplexsonographie, um pathophysiologische Zusammenhänge interessant und bildhaft darzustellen. So können pathologische Befunde durch die Studierenden nach der Primäruntersuchung schnell und ohne viel Aufwand selbständig mit dem Schallkopf in der Hand am Patienten nachvollzogen werden. Die ersten Schritte im Operationssaal sind für die Studierenden meist mit viel Aufregung verbunden, da die vielen geordneten Abläufe und Regeln schnell das Risiko bergen, zu Negativerfahrungen führen zu können. Hier gilt es, die Studierenden nicht zu überfordern, um ihnen nicht das Gefühl zu geben, an einem schwierigen Operationsverlauf (mit) schuld zu sein. Vielmehr gilt es, die vielfältigen Möglichkeiten und Varianten der chirurgischen Operationsabläufe aufzuzeigen. Hier eignet sich eigentlich besonders die Gefäßchirurgie, da die Operationen in der Kombination von offener und endovaskulärer Therapie in der Lage sind, die Therapie an die Patientinnen und Patienten individuell anzupassen und Revaskularisationsbemühen und -ergebnis mithilfe der DSA zu visualisieren.

### Praktisches Jahr („PJ“)

Das PJ bildet in der humanmedizinischen Ausbildung den Höhepunkt. Hier soll zum ersten Mal das erworbene Wissen kontinuierlich am Patienten unter Anleitung angewendet und vertieft werden. Vor dem Hintergrund der steigenden Patientenzahlen, der zunehmenden Bürokratie und des ärztlichen Personalmangels lief das PJ Gefahr, in der Ausbildung vernachlässigt zu werden, sodass die Studierenden oft nur mit Anamneseerhebung und Blutentnahmen beschäftigt waren. Mit dem allgemeinen Mangel an Nachwuchs in der Chirurgie wuchs auch das Bewusstsein bei den Lehrenden und verantwortlichen Ärzten, die Studierenden für eine Ausbildung in der Chirurgie frühzeitig zu interessieren und das PJ durch Struktur, Lernziele und direkte Anleitung zu verbessern. Die „Chirurgische Arbeitsgemeinschaft Lehre“ (CAL) sieht den patientennahen Kompetenzerwerb während der 12 Wochen des chirurgischen Pflichtquartals bzw. 16 Wochen des chirurgischen Pflichttertials weiterhin im Vordergrund stehen [17]. Um das PJ in den chirurgischen Fächern an der Medizinischen Fakultät mit „Universitätsklinikum Magdeburg A.ö.R.“ zu strukturieren, wurde ein chirurgisch determiniertes PJ-Logbuch unter Berücksichtigung der diversen chirurgischen Fachdisziplinen mit ihren besonderen Stärken erarbeitet. Damit soll sichergestellt werden, dass die PJlerin und der PJler selbstbestimmt, aber doch strukturiert und arbeitsbegleitend die unverzichtbaren chirurgischen Kenntnisse und chirurgischen Fähigkeiten erwerben kann [18]. In dem Logbuch können die erlernten Fähigkeiten und Kenntnisse eigenverantwortlich dokumentiert und strukturiert abgearbeitet werden. Es repräsentiert damit die chirurgische Lernbiografie und kann auch als Vorzeige- oder Bewerbungsportfolio benutzt werden sowie dient dem kompetenten Nachweis einer adäquat erfolgten Erfüllung der Lehrvorgaben und effektiven Prüfungsvorbereitung [18]. Die Erfüllung der Lernziele bleibt dabei in der Hand der PJlerin und des PJlers und hängt nicht zuletzt von deren/dessen Motivation und Initiative ab, jedoch ist hier eine Struktur und Übersicht über die chirurgischen Lernziele vorhanden. Es wird auf eine 60 % ige Erfüllung orientiert. Die Ausbildungsinhalte wurden anhand des PJ-Basis-Logbuchs des „Medizinischen Fakultätentages“ erarbeitet und in Tabellenform mit der Möglichkeit der Dokumentation verfasst. Die Dokumentation erfolgt in den Sparten: 1. theoretische Kenntnisse, 2. gesehen, 3. selbst unter Anleitung ausgeführt und 4. Beherrschung (selbständige Ausführung ohne Supervision). Ähnlich verhält es sich bei der Erarbeitung der Krankheitsbilder, die in der Form

- theoretische Kenntnisse, demonstriert oder gesehen (und)
- klinischer Verlauf mehrtägig verfolgt,

dokumentiert werden. Die demonstrierten und assistierten Operationen werden ebenfalls benannt. Abschließend wird der PJlerin und dem PJler in einer klinikinternen Falldemonstration die Möglichkeit gegeben, einen ausgewählten klinischen Fall zu präsentieren, um die erlernten Kenntnisse zu diskutieren und zu vertiefen.

## Diskussion

Die Entwicklung der gefäßchirurgischen Lehre an den Universitäten innerhalb des Humanmedizinstudiums ist ein anhaltendes Erfordernis vor dem Hintergrund

- eines stetig wachsenden Wissenszuwachses im Fachgebiet Gefäßchirurgie,
- des zu erwartenden Anstiegs der Patientenzahlen in den nächsten Jahren mit notwendigem Ausbau der Behandlungskapazitäten (und)
- des zu erwartenden Mangels an dem Fachgebiet interessierter Studierender.

Nicht zuletzt sind daher die Bemühungen der Fachgesellschaft mit zahlreichen Publikationen zu den Möglichkeiten der Lehre in der Gefäßchirurgie und der Strukturierung der Ausbildung als Reaktion auf diese Erfordernisse zu werten.

In erster Linie bleibt die Gefäßchirurgie in der Lehre an die Allgemeinchirurgie durch eine abgestimmte Organisation der Vorlesungen, Seminare und Praktika gebunden. Zu fordern sind jedoch eine ausreichende Anzahl an akademischen Lehrstunden und die adäquate Verteilung von Kapazitäten zum Training praktischer Fähigkeiten und der fallbezogenen Lehre am Krankenbett. Voraussetzung dafür ist dabei das Vorhandensein des notwendigen akademischen und lehrinteressierten, ja – enthusiastischen Lehrpersonals. Damit verbunden ist die Förderung der Bildung weiterer gefäßchirurgischer Lehrstühle, um die Gefäßchirurgie aus ihrem Sektionsdasein in klinischer und akademisch wissenschaftlicher Hinsicht herauszuholen [6]. Die Gefäßchirurgie muss für den Humanmedizinstudierenden sichtbarer und präsenter werden.

Bislang gibt es zu wenige eigenständige universitäre gefäßchirurgische Standorte mit der Möglichkeit zur wissenschaftlichen Arbeit und akademischen Lehre. Neben dem Erhalt dieser Lehrstühle ist die Neuetablierung eigenständiger Kliniken zu fördern, um mit eigenen Ressourcen und Formaten die gefäßchirurgische Lehre weiterentwickeln zu können. Vor diesem Hintergrund wurde von der DGG die Exzellenzakademie zur Identifikation potenzieller Kandidaten sowie zur Ausbildung und Unterstützung bei der Bewerbung auf akademische Chefarztstellen gegründet [19].

Die Möglichkeiten der gefäßchirurgischen Lehre sind durch die Einführung des Skills-Lab-Trainings oder extrakurrikularer (fakultativer) Lehrformate in den letzten Jahren gewachsen, was nicht zuletzt eine immense Ausweitung des Lehraufwandes durch die Lehrverantwortlichen bedeutet, aber auch die Chance einer bedeutsamen Aufwertung des Faches bietet. Nicht zuletzt wird ggf. aufgrund von Personalmangel die Lehre im Skills Lab, Blockpraktikum oder in extrakurrikularen Veranstaltungen an Assistenzärzte weitergegeben, die nebenher noch die alltägliche Stationsroutine ableisten müssen. Somit hängt trotz aller technischen und multimedialen Möglichkeiten die Qualität der Lehre von entsprechend freigesetztem, gut vorbereitetem und engagiertem Lehrpersonal ab. Auch wenn von universitären Ärzten ein Beitrag zur Lehre zu erwarten ist, wird die dadurch entstehende Doppelbelastung selten honoriert oder vergütet. Somit klafft zwischen Wunsch und Wirklichkeit häufig eine Lücke, die es zu schließen gilt. Insofern ist die kompetente Anwendung und konsequente Umsetzung des NKLM eine wichtige Voraussetzung, eine strukturierte Lehre in der Gefäßchirurgie zuverlässig und nachhaltig zu gewährleisten. Die Standardisierung der Lehrinhalte ermöglicht dem Lehrteam, eine Basis für die Ausbildung zu schaffen. Dies ist insbesondere in Zeiten notwendig, in denen nur eine Onlinelehre möglich ist. Die Herausforderungen in den letzten fast zwei „COVID-Jahren“ waren enorm. Abgesehen von den technischen Schwierigkeiten war die Erstellung neuer Lernformate und deren Visualisierung in Lernplattformen zur selbständigen Erarbeitung durch die Studierenden eine Herausforderung [20], während für die Studierenden der Umgang mit den neuen Medien eher schon die Routine ist. Dies eröffnet zudem die Möglichkeit, den Studierenden die Wissensvermittlung im Wettbewerb zur Frontalvorlesung im Hörsaal auf andere Weise „schmackhaft“ zu machen, wobei sich die konventionelle Vorlesung mit vorwiegend und bestimmend direktem Lehrer-Studierenden-Kontakt nach Beendigung der Corona-Pandemie wieder durchsetzen sollte. Der Erwerb spezifisch chirurgisch-motorischer Fähigkeiten durch Hand-Auge-Koordination, Wiederholung und Training ist trotz multimedialer Möglichkeiten eine ganz entscheidende und spezifische Stärke der direkten chirurgischen Lehre.

Die Strukturierung des PJ mit Implementierung des PJ-Logbuchs bietet die Möglichkeit für Studierende und Lehrende, die Wissensvermittlung und den Erwerb der praktischen Fähigkeiten genau zu dokumentieren, zu überprüfen und von beiden Seiten einzufordern. Denn gerade im PJ ist die Umsetzung und Anwendung des erlernten Wissens sowie der Ausbau der praktischen Fähigkeiten unter Anleitung durch einen strukturierten Plan effektiv umsetzbar. Die Gefäßchirurgie eröffnet durch die Multimorbidität der Patienten die Möglichkeit, die medizinischen Kenntnisse und praktischen Fertigkeiten fachübergreifend und umfassend anzuwenden. So bietet allein die Behandlung chronisch infizierter Wunden bei pAVK-Patienten neben der entsprechenden stadienadaptierten Wundtherapie den Ansatz, initiale bis ausführliche Erfahrungen auf dem Gebiet der Diabeteseinstellung, Behandlung einer Herz- und Niereninsuffizienz, Medikamenteninteraktion, der Krankenhaushygiene, Antibiotikatherapie (inkl. Resistenzentwicklung) und operativ-chirurgischen Techniken der Amputation im Setting des perioperativen Managements zu erwerben. Damit lernt der Studierende, wie wichtig es ist, fachübergreifend und teamorientiert zu arbeiten, um die komplexe Erkrankung einer pAVK zu beherrschen.

## Zusammenfassung

Die Lehre in der Chirurgie/Gefäßchirurgie wird sich in den nächsten Jahren mit multimodalen und -medialen Angeboten, mehr praxisorientierten Anteilen sowie mit fortgeführter und weit intensiverer Integration der Studierenden in den Klinikalltag den Herausforderungen des exponentiellen Wissenszuwachses, der rasanten technologischen Entwicklung und dem zunehmenden Arbeitspensum als auch den Studierendenforderungen stellen müssen. Voraussetzung dafür ist die Aufwertung der Gefäßchirurgie durch strukturelle Veränderungen an den Universitätskliniken, der Einsatz didaktisch geschulter Lehrender mit entsprechender fachlicher Expertise und entsprechenden organisatorischen Freiräumen sowie Ausbau der technischen Erfordernisse an eine multimediale Lehre. Der „Nationalen kompetenzbasierten Lernzielkatalogs Medizin“ (NKLM) bildet die Grundlage für das zu erlernende Wissen; die praktischen Erfahrungen und Fertigkeiten erlernen die Studierenden jedoch nur unter fachlicher Anleitung kompetenter und lehraffiner Dozentinnen und Dozenten sowie engagierten AssistentInnen bei intensiver Integration in den Praxisalltag.
